# Open-access healthcare debriefing videos need to incorporate more Safety-II learnings

**DOI:** 10.1186/s41077-025-00345-3

**Published:** 2025-04-14

**Authors:** Suzanne Bentley, Alexander Meshel, Komal Bajaj

**Affiliations:** 1 New York City Health + Hospitals/Elmhurst, Elmhurst, USA; 2 Icahn School of Medicine at Mount Sinai, New York, USA; 3 Mount Sinai Hospital, New York, United States; 4 New York City Health + Hospitals/Jacobi, Bronx, USA; 5 Albert Einstein College of Medicine, Bronx, USA

**Keywords:** Debriefing, Safety, Safety-II, Simulation

## Abstract

**Background:**

Patient safety science and debriefing approaches have historically tended to focus most heavily on Safety-I or “why things go wrong” and learning from unfavorable performance, root cause of adverse outcomes, and improvement opportunities learned from failures. Consequently, rich opportunities for analysis and learning from “why things go right,” successful performance, and exploration of how systems succeed, adapt, and perform effectively regardless of outcome—Safety-II—are often underrepresented.

**Methods:**

Open-access videos of healthcare debriefing were sought by searching Google and YouTube via search terms “healthcare debriefing,” “healthcare debrief,” “healthcare debriefing video,” “healthcare debrief video,” “healthcare debriefing example,” “healthcare debrief example,” “simulation debriefing,” and “simulation debrief.” Additionally, a search of major professional organization websites was utilized. Included videos were reviewed to score all utterances on the following: (1) phase of debriefing; (2) question or statement; (3) by facilitator or participant; (4) if utterance was neutral, related to positive performance/ “what went well” or negative performance/“what could be improved”; (5) if facilitator utterance was general or a follow-up, reflective utterance building upon previous discussion; (6) if participant utterances were general or specific reflective, insight offering comments; (7) all facilitator follow-up/ specific reflective type utterances were further analyzed and coded as exploration into Safety-I (e.g., exploration of why error occurred) or Safety-II (e.g., adaptability, variation, reproducing success) concepts.

**Results:**

A review of open-access video examples of healthcare debriefing demonstrates disproportionate emphasis on Safety-I and highlights the opportunity for open-access examples of healthcare debriefing to include additional language and techniques that promote and role model inclusion of Safety-II discussion.

**Conclusions:**

While there is always room for improvement and we must all strive to do the best we can, we are missing a major opportunity to build resilience by Safety-II exploration into analyzing why things go positively. Those designing such instructional videos should intentionally include debriefing focused on both Safety-I and Safety-II aspects of performance, regardless of outcome, as they are both important, complimentary, and result in a more holistic understanding of improvement opportunities and success. Future study on the impact of Safety-II debriefing should focus on context-specific promotion of quality and patient safety, as well as impact on participant wellbeing and overall safety culture.

## Background

Healthcare is inherently a safety critical industry [1–3] and the daily work performed by healthcare professionals and teams impacts patient outcomes—favorably or unfavorably. The seminal 1999 Institute of Medicine report “to Err is Human” is often credited for catalyzing efforts to study and improve safety in healthcare [4]. The report highlights the immense toll of preventable medical errors in hospitals and asserted the “problem is not bad people in healthcare- it is that good people are working in bad systems that need to be made safer” [4, 5].

Historically, professionals in safety critical industries have focused primarily on reducing risk and minimizing harm by learning everything possible from when things go wrong, referred to as Safety-I. Despite tremendous investment in this strategy, the outcomes are disappointing. Safety science continues to evolve and the limitations of a Safety-I focused strategy are becoming clearer with a growing imperative to expand our thinking and practices [6]. In contrast, Safety-II encourages the study of all events, regardless of outcome, and includes review of routine and mundane cases as well as rare and exceptional cases [7–9].

Debriefing is one important tool to study events. The Agency for Healthcare Research and Quality (AHRQ) defines debriefing as a “directed, intentional conversation that can be used for knowledge or skill attainment, or to answer questions about threats to patient safety and patient care based on a recent event or a hypothetical situation ” [10]. Debriefing is routinely utilized following a simulation and debriefing after actual clinical cases is recommended after all significant clinical events to make improvements in individual, team, and system performance [11, 12]. Studies show improved effectiveness in teams that debrief following cardiopulmonary resuscitation events compared with those that do not, as well as measurable improved clinical outcomes, such as achieving return of spontaneous circulation and neurologic outcomes [13]. One debriefing study of utilization of debriefing checklists in the operating room after all surgical cases resulted in debriefing-driven improvements such as better workforce safety culture, lower costs, reduced proportion of surgical cases with reported defects, and significant reduction in the 30-day unadjusted surgical mortality [14]. Additionally, studies of the impacts of debriefing reveal improved interdisciplinary practice, teamwork and communication, error reduction, enhanced skill development, and emotional support benefits to participants [15–21].

Debriefing and learning from success is not uncommon or novel; however, similar to safety science’s historic emphasis on Safety-I concepts, many common debriefing strategies also appear to focus most heavily on Safety-I [22, 23]. Expansion to include more formal emphasis on Safety-II concepts would provide powerful analysis of everyday work and allow for expansion from the more traditional Safety-I focus on error analysis and “what went wrong” or “could have gone better” to also capture analysis of Safety-II and concepts such as capacities, adjustments, variation, and adaptation for successful operations in a complex system [9, 24]. Debriefing inclusive of a more Safety-II focus acknowledges that there are important opportunities for learning and improvement from all events, regardless of outcome [22, 25]. Additionally, its inclusion of focus on positive performance, which may appear to be “lower stakes” for participants to debrief, may encourage and socialize increased debriefing performance, overall.

To further investigate the hypothesized disproportionate focus on Safety-I over Safety-II in healthcare debriefings, the objective of this study was to assess and quantify the inclusion of Safety-II discussion in open-access post-simulation debriefing videos on the internet.

## Methods

Open access videos of healthcare debriefing were sought by searching Google and YouTube via search terms “healthcare debriefing,” “healthcare debrief,” “healthcare debriefing video,” “healthcare debrief video,” “healthcare debriefing example,” “healthcare debrief example,” “simulation debriefing,” and “simulation debrief.” Additionally, videos were sought via search of major professional organization websites (e.g., American Health Association, AHRQ, American Hospital Association, American College of Emergency Physicians, American College of Obstetrics and Gynecology, American College of Physicians, American Medical Association, American College of Surgeons, Royal Australasian College of Physicians, Royal College of Surgeons) for open-access videos of healthcare debriefings.

Available healthcare videos were identified from these sources and screened with the inclusion of videos from consortiums and professional organizations and the exclusion of single sites (e.g., video created by single hospital-based simulation center). Single-site debriefings, while valuable, reflect a narrower, more localized perspective that may not be as generalizable to the wider population of hospitals or debriefing settings vs. consortium or professional organization content incorporating more comprehensive and diverse sets of perspectives from across institutions. Next, videos were reviewed for eligibility with the inclusion of videos if they contained debriefing discussion of a specific case with details on specific case and discussion of team’s or individual performance. All other videos were excluded, such as those providing general discussion of healthcare debriefing, review of or demonstration of steps in debriefing but not a debriefing itself, and critical stress event debriefing videos focused solely on emotional processing without case review.

Those 7 remaining videos were watched in full to ensure adequacy of the accompanying transcript from YouTube and any annotations required for clarity were made to transcript to allow for full review and analysis of comments. Transcripts were then formatted to separate individual utterances (discreet statements/questions) into separate lines for review and each was marked to which phase of debriefing during which it occurred (mapped to PEARLS debriefing tool) [26]. Videos and accompanying formatted transcriptions were reviewed by 2 independent reviewers to score all utterances. Attributions were compared and discussed until agreement, if initial disagreement on any of the initial responses between reviewers.

While discerning focus on Safety-I or Safety-II in the videos, we first assessed the overall proportion of focus on positive versus negative performance. Safety-II includes important focus on understanding positive performance, “learning from success,” and inclusion of analysis of what went “right” and why. Acknowledging, however, that Safety-II includes far more than a simple focus on review of “what went well,” we next assessed utterances to discern those that were more initial comments or general, unanalyzed utterances versus reflective, analytical, deeper dive utterances. Facilitators’ reflective, analytical or deeper dive discussion points, whether focused on positive or negative performance aspects, were then assessed to determine if their focus mapped to Safety-I (e.g., error identification, root cause analysis, compliance with standard protocols) or Safety-II (e.g., reproducing success, variability, adaptation) type exploration. This allowed for further insights and differentiation of utterances between general review of positive performance and specific Safety-II concept exploration, when possible. Additionally, this ensured capture of Safety-II concepts explored in the discussion of both negative or positive performance aspects.

As illustrated in Fig. 2, debriefing utterances during analysis phase were categorized as (1) made by facilitator or participant; (2)question or observation/statement; (3) if utterance was neutral, related to discussion of positive performance and “what went well” or discussion of negative performance and “what could be done differently” or improved; (4) if facilitator utterances were general or a stated observation (e.g., “tell me more”; “I saw you started an IV”) or follow-up comment building upon previous discussion point (from facilitator or participant) or offering specific insight or observation provided with analysis of its significance (e.g., “you called for help and I was curious why you called out into the hallway and did not use call bell); (5) if participant utterances were general, stated observations (e.g., “the patient looked sweaty”) or offering specific insight or observation with analysis of its significance (e.g., “the patient looked sweaty so I started to think what is the past medical history and that then led me to quickly check the blood sugar”); (6) All follow-up or specific insight type utterances were further analyzed and coded as focus on Safety-I (e.g., exploration of why error occurred) or Safety-II (e.g., adaptability, variation, reproducing success) concepts, when applicable. Of note, Safety-II discussion points were captured from debriefings regardless of positive or negative performance or outcome, when applicable, highlighting Safety-II emphasis on learnings from all case types.

## Results

**Fig. 1:**
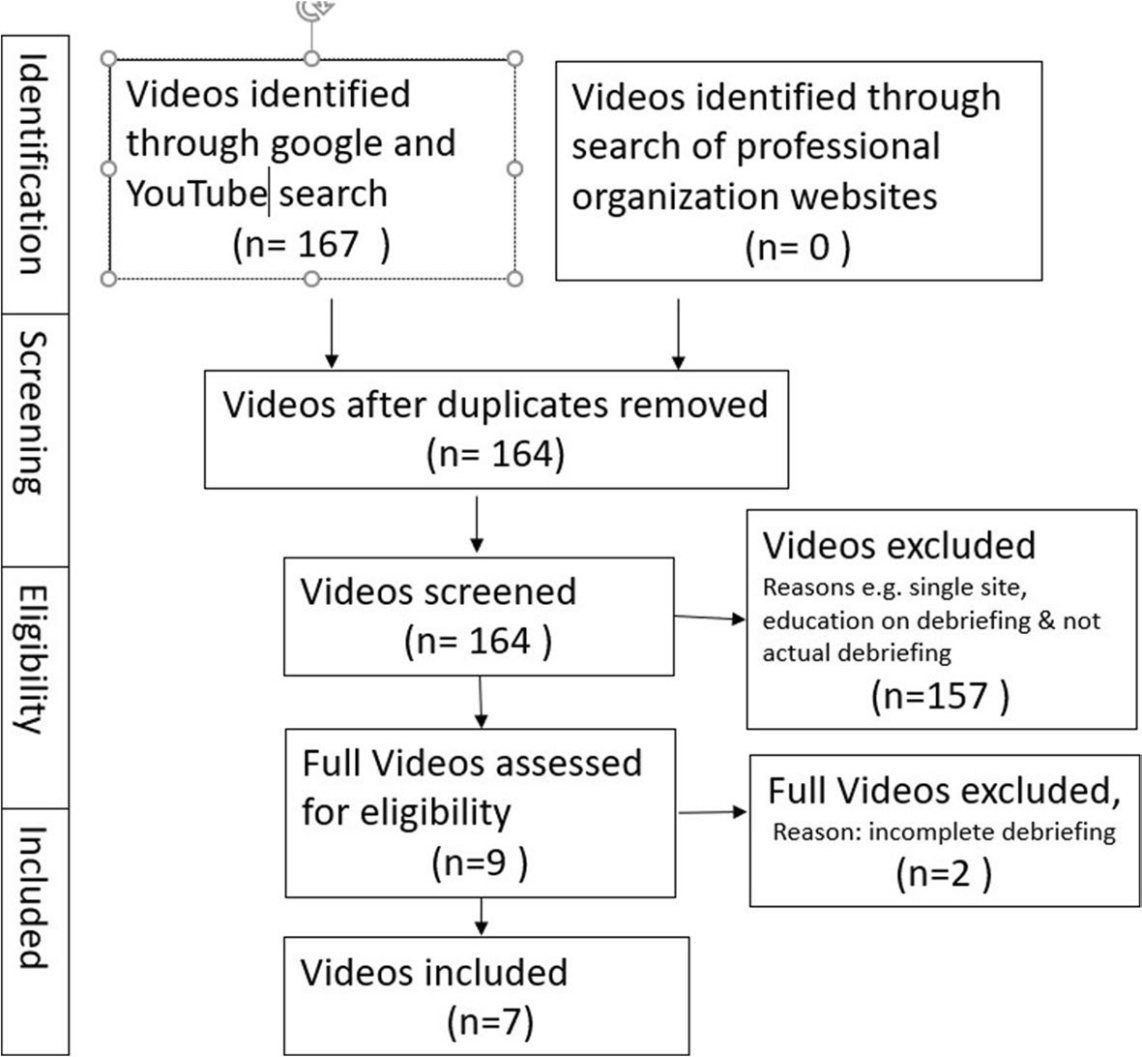
Open access healthcare debriefing video screening and inclusion

Seven videos met inclusion criteria and these videos and transcripts were reviewed and analyzed (Fig. 1). These videos were from 6 discreet organizations (with inclusion of 2 separate debriefing videos from the AHRQ). These videos were intended to be instructional and to serve as models of healthcare debriefing excellence. They each included a healthcare debriefing after simulated clinical case. The video case types debriefed included a variety of cases, such as evaluation and management of confusion in setting of hypoglycemia, cardiac arrest resuscitation, and respiratory compromise in patient with obstructive lung disease.

In the analysis phase of these 7 videos, the facilitators in total asked 24 questions and made 30 statements and the participants asked 1 question and made 59 statements. Table 1 displays a breakdown of utterance type and focus on negative or positive performance. During the analysis phase, 29% of the facilitators’ questions referred to negative performance aspects, 25% to positive performance, and 46% were neutral; with the majority being statements following the plus/delta format [27] of “what went well?” as most general exploration of positive behavior or attempting to learn from success and “what could have been done differently?” as negative or improvement exploration. Neutral utterance examples include “let’s unpack the scenario”, “who wants to share more?” and “please share how you think the case went” Of facilitator statements, 50% related to negative aspects of performance, 43% to positive performance, and 7% neutral. Negatively focused examples included “what didn’t go so well?”; “why did you not call pharmacy when medication wasn’t sent up?”; “you noticed patient was hypoxic but did not place oxygen or address it.” Positively focused examples include “what went well?” Notably, that question nor others were ever followed up with any variation of “why did it go well?” or further exploration. Other positive focused examples include: “you got supplies to bedside”; “your assessment was very prompt and I did hear you say that he’s getting really sweaty”; “I noticed the team paused to regroup.”

**Table 1:** Debriefing analysis phase total utterances (questions or statements) by facilitator vs. participant; total # and # neutral or referring specifically to an aspect of positive/(+) performance vs. negative/(−) performance

	Facilitator questions: total *N* = 24			Facilitator statements: total *N* = 30			Participant questions: *N* = 1	Participant statements: total *N* = 59		
Type	(−)	(+)	Neutral	(−)	(+)	Neutral	Neutral	(−)	(+)	Neutral
Total	7	6	11	15	13	2	1	18	41	0
%	29%	25%	46%	50%	43%	7%	100%	31%	69%	0

Of participant statements, 31% were related to negative performance and 69% focused on positive performance. Examples related to negative performance were “I didn’t call for more help”; “I didn’t notice the change in oxygen levels”; “the rate of compressions was off.” Examples of positive performance were “we started CPR right away”; “we worked together.”

As shown in Table 2, of all negative behavior-focused facilitator statements, 73% (11 of 15) were follow-up, specific, and deeper exploration type statements compared to 27% (4 of 15) general or non-specific statements. In contrast, of all positive behavior focused facilitator statements, 31% (4 of 13) were follow-up, specific, and deeper exploration type statements compared to 69% (9 of 13) which were general or non-specific statements.

**Table 2:** Analysis phase total utterances, number and percentages of general utterances vs. specific, reflective or follow-up utterances of facilitator vs. participant for positive or negatively focused utterances

	Negative/(−) facilitator statements. Total = 15		Positive/(+) facilitator statements. Total = 13		Negative/(−) participant statements. Total = 18		Positive/(+) participant statements. Total = 41	
Type	Specific	General	Specific	General	Specific	General	Specific	General
Total	11	4	4	9	16	2	13	28
%	73%	27%	31%	69%	89%	11%	32%	68%

**Fig. 2:**
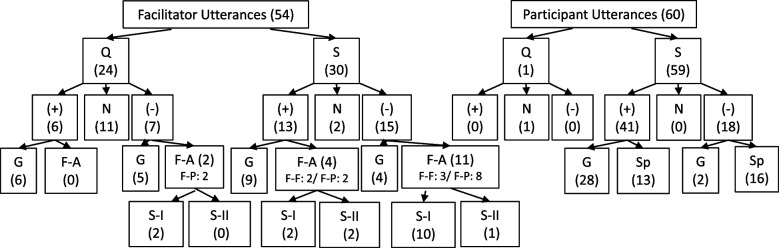
Flow of utterance analysis with corresponding numbers of occurrence of each in parentheses. Q, question; S, statement; (+), positive performance-focused utterance; (−), negative performance-focused utterance; N, neutral; G, general, nonspecific or initial utterance; F-A, follow-up or analytic utterance, intended to expand discussion; F-P, facilitator follow-up to participant utterance; F-F, facilitator follow-up to facilitator’s own utterance; S-I, Safety-I; S-II, Safety-II; Sp, specific content utterance

**Fig. 3:**
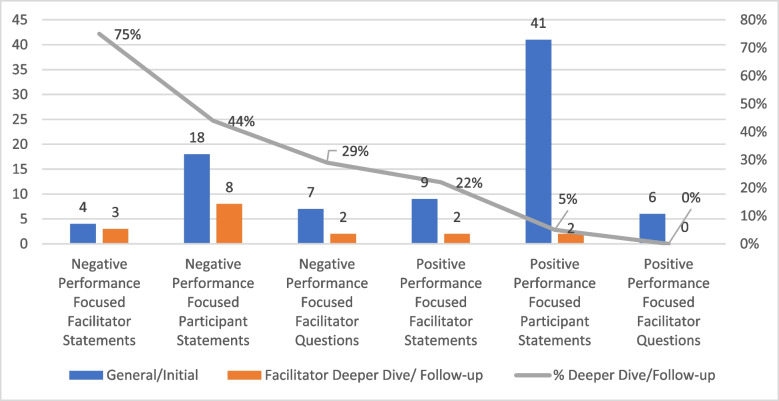
Follow-up or expanding explorations by facilitator after facilitator and participant negatively focused and positively focused utterances in analysis phase of debriefing

As shown in Figs. 2 and 3, facilitators offered follow-up or additional discussion statements to their own initial or general statements with a negative focus 75% of the time, compared to follow-up utterances after 25% of their initial positively focused statements. Also, facilitators offered follow-up or deeper dive discussion to participants’ negatively focused statements 44% of the time, compared to 5% of the time when positively focused statements were made by participants. Examples of positively focused follow-up statements include “using SBAR helped communicate the situation easily” (to participant who initially stated “communication seemed to be good and we had hand-off”); “when you call each other by name we are able to know what everybody is doing” (to initial facilitator statement “teamwork went well and roles seemed clear”). Negatively focused examples include “you didn’t place a definitive airway despite the ongoing hypoxia” (to participant’s initial statement “I noticed the change in oxygen levels”); “you knew labs were needed but no one set up for it” (to participant statement “we never drew labs”). Similarly, facilitators offered follow-up questioning more often to negative discussion points than positive (29%, 0%, respectively).

Notably, participant debriefing statements favored discussion of positive performance aspects overall (69%); however, only 32% (13 of 41) of the positively focused statements were specific, analytical comments vs. 68% (28 of 41) general or statement of observed performance without elaboration on context or significance of statement being shared. In contrast, 31% of overall participant statements were focused on negative performance aspects, but 89% (16 of 18) of the negatively focused statements were specific, analytical comments compared to 11% (2 of 18) general or statement of observed performance without elaboration on context or significance of statement being shared.

Highlighted in table 3, upon further analysis of facilitator follow-up, deeper dive type utterances, the negative behavior-focused statements by facilitators revealed 91% (10 of 11) mapped to Safety-I exploration and 9% (1 of 11) which mapped to Safety-II exploration. In contrast, of the positive behavior-focused follow-up/deeper exploration type statements by facilitators, 50% (2 of 4) mapped to Safety-I exploration and 50% (2 of 4) mapped to Safety-II exploration. Safety-II-related discussion comments were further analyzed to map to specific Safety-II-related concepts (e.g., reproducing success, variability, adaptation), differentiating between the concept of learning from success via general review of positive performance (“what went well?”) and specific Safety-II concepts, when possible. [22]

**Table 3:** Analysis phase total utterances, number and percentages of Safety-I vs. Safety-II vs. Safety-II exploration in follow-up, specific, reflective utterances of facilitator for positive or negative focused utterances

	Specific negative/(−) facilitator statements. Total = 11		Specific positive/(+) facilitator statements. Total = 4	
Type	Safety-I	Safety-II	Safety-I	Safety-II
Total	10	1	2	2
%	91%	9%	50%	50%

The most common Safety-II concepts explored were discussions surrounding learning from and reproducing success and “how can we ensure we do this again in the future?” and “discussion of why something positive occurred.” Additional and more specific Safety-II concepts highlighted in analysis of the video transcriptions include:

- Discussion of successful adaptation (e.g., teamwork to find alternate solutions when initial steps not successful)
- Exploring variability
- Flexibility
- Adaptive capacity (e.g., meeting demands of situation despite resource limitation)
- Comparing cases (past to present)
- Exploration of aspects of work as done vs. work as imagined.

Some discussion examples mapped to multiple Safety-II concepts given the interrelated nature of certain concepts. It is important to note that these terms were assigned when the debriefing material was analyzed and concept mapped for the purposes of this study and that none of these terms were explicitly named despite being explored. Majority of points mapped to Safety-I concepts included identifying errors, root cause analysis of negative performance, delays in care, failure to escalate, compliance with standardized procedures, and deviations from expected performance or standardized protocols.

Additionally, 3 of the 7 video debriefings included a summary phase of debriefings, which were analyzed. In total, 83% of the facilitators’ summary comments highlighted Safety-I performance-focused statements (all focused on negative performance aspects in the cases), with 5 Safety-I-focused utterances and a single Safety-II-focused statement. Participants contributed 3 Safety-I statements and 0 positive. The Safety-II focused comment was “it’s important to continue to use communication tools” (reinforcing positive performance because these tools were utilized during the case). Examples of Safety-I summary points include “remember you’re never alone and to call for help, whether it be to call an attending or a call to a colleague” (reminder of required future action after negative performance because help was not obtained during the case); “patients in distress need oxygen including those that have COPD” (reminder to not repeat error made in specific patient in the case who was not placed on oxygen). Of note, in the analysis phase of these 3 videos, a total of 8 positive and 7 negative facilitator utterances were made and 14 positive and 9 negatively focused participant utterances were made.

## Discussion

In a debriefing, it’s crucial to differentiate between general/nonspecific and specific reflective comments, as nonspecific comments often do little to further the discussion or analysis, while specific, analytic or reflective comments serve to deepen or broaden the discussion, and ultimately learning. A debriefing facilitator plays a pivotal role in encouraging participants to dive deeper into their comments, asking probing questions, and guiding them to reflect more meaningfully on their thoughts. What the debriefer chooses to focus on and dive more deeply into discussing ultimately drives the direction of the debriefing and thereby the focus of the lessons learned.

Facilitators followed up their own statements much more often when focused on negative performance (75% vs. 22%), admittedly with small overall number of follow-up utterances offered in the data set. More revealing, however, is analysis of the facilitator follow-up utterances to participants’ statements that showed facilitators continued or broadened discussion after 44% of negative performance-focused statements by participants compared to after just 5% of positively focused statements. Keeping in mind that participants in these specific videos offered more than double positive to negative performance comments to discuss, facilitators still followed up negative performance discussion more often (4 positive and 8 negative follow-ups vs. 41 positive and. 18 negative participant statements). Facilitators failed to meaningfully follow-up on the majority of the positively focused participant statements.

Facilitators and participants both favored deeper, reflective discussion of negative performance aspects. Following similar trend to the facilitators, analysis of participant utterances as general/nonspecific or specific, reflective, or deeper dive type utterances revealed 89% preponderance of follow-up or reflection type utterances when discussing negative performance aspects compared to 11% general negative focused utterances. In contrast, only 32% of participants’ positively focused utterances were follow-up or deeper reflective type utterances, compared to 68% general, nonspecific positively focused questions. This reveals that while participants offered substantially more discussion of positive performance overall (69% vs. 31%), they lacked in specificity and reflection on majority of the positive focused aspects, instead offering mostly stated observations of “what went well” without context or significance, never broadening to analyze “why it went well.” In contrast, despite fewer overall negative utterances, participants were more specific and analytic in their discussion of negative performance aspects. Ultimately, they offered 16 specific negative performance aspect comments and 13 positives, despite less than half of their overall utterances being negatively focused compared to positively focused.

Ultimately, facilitators drive the discussion direction and what is included, as well as setting the set the tone for the expected focus of discussion. It is therefore not surprising that participants reflected on and further analyzed more of the negatively focused discussion points, matching the facilitators’ disproportionate emphasis on negative performance in the debriefings. This focus is further reinforced in the debriefings’ summary phases that served to disproportionally reinforce learning points from negative performance and what not to repeat in the future, without similar attention to learning points from positive performance and successes in the cases.

Addressing our stated objective, the facilitators’ specific, reflective utterances were mapped to a focus on Safety-I (e.g., error identification, root cause analysis, compliance with standard protocols) or Safety-II (e.g., reproducing success, variability, adaptation) type exploration. The overwhelming majority (91%) of all negatively focused specific or follow-up utterances by facilitators favored Safety-I exploration, with the inclusion of 1 exploration of Safety-II. Of all that were positively focused, however, 50% explored Safety-II and 50% explored Safety-I. It is not surprising that most review of negative performance aspects was mapped as exploration of Safety-I. It is beneficial that 1 instance was broadened to explore Safety-II, as well. However, of all of the explorations of positive performance (of which there were far fewer than explorations of negative to start), only 50% included exploration of Safety-II, with the other 50% resorting back to otherwise noted emphasis on exploration of error identification and prevention, Safety-I.

The emphasis on questioning and discussing negative aspects of performance and focus on Safety-I in debriefing is not necessarily surprising since historically safety science has predominantly emphasized and thereby perhaps biased or primed us to more commonly seek opportunities to learn from failure. Additionally, many common debriefing strategies appear to focus most heavily on Safety-I [23]. This analysis of open-access healthcare debriefing examples was conducted to attempt to quantify this phenomenon, one that we do not think will surprise nor necessarily contradict what many educators and quality and safety experts observe in practice.

There are concrete examples of learning from success facilitated by further deep-dive discussion into aspects of performance, both negative and positive, from a Safety-II lens. As outlined above, specific Safety-II concepts were noted in the analysis of the videos. For example, a Safety-II lens was applied in one debriefing of a robust discussion on why cardiopulmonary resuscitation (CPR) compressions were completed with accurate timing (positive outcome). The facilitator asked a participant about CPR saying, “did you get tired or have trouble with the pace of compressions?”. The participant responded that “I was starting to slow down but Shelly was able to correct the rate of compressions so I was able to stay on pace, so having someone pace you through codes is helpful.” In this example, the facilitator and team agreed compression pace was appropriate and participant identified an “aha moment” of thinking more deeply about the why of the positive performance—appropriate compression rate in this example. Subsequently, a second participant suggested piloting the use of a metronome in their clinical area and the facilitator agreed to raise this idea to leadership for future discussion. This discussion of why the performance was positive was followed by brainstorming on ways to reproduce or ensure future positive performance and highlights the learning opportunity from something that otherwise may not have been discussed because “it went well.”

In a debriefing of a specific respiratory case that did not proceed smoothly, had a poor outcome, and multiple negative performance aspects, Safety-I discussion ensued but also included exploration of Safety-II exploration. Negative performance included delays to recognition of deterioration and initiation of resuscitation. Safety-I analysis including listing the errors made, discussing why the team thought the delays occurred, and discussing how their performance did not adhere to resuscitation guidelines. However, discussion is broadened by the facilitator who asks “how was it different with other patients you’ve had with this [condition]? Why did a [previous] case go well instead?”. This questioning serves to explore variability and conditions or resources promoting/hindering success; allows for comparison of cases (present with past); and discussion of positive performance in other previous cases to inform learning during the present negative performance. Additionally, a team member noted how helpful it was another team member suggested a certain alternate procedure when the team was struggling with initial procedure (placing interosseous line when intravenous line was difficult). The discussion reinforced how the team was successful with this step in the resuscitation because they were responsive to each other, solicited and accepted alternate suggestions when initial attempts were not working, and that this was an appropriate and successful adaptation, which should be considered more quickly in the future in a similar case. This particular video did not include a summary phase but we hope key takeaways for reinforcement would’ve been these aspects of reproducing success.

Conversely, in a different case with positive outcome, which met objectives and included numerous positive performance aspects, the debriefing focus shifted to Safety-I, without the inclusion of Safety-II. During the case, a participant called the nurse to verbally request a medication (which was then given). In the debriefing, the participant shared “I should have written down the order or entered a standing order.” Despite the positive outcome and use of different, allowed ordering strategies, this debriefing discussion focused most on Safety-I and procedural improvement and what could have been done differently to adhere to standard protocols. It missed the opportunity to explore a Safety-II focus, such as broadening the discussion to reinforce the flexibility and quick action taken to call the nurse instead of time to place a computer order during emergency scenario and how that contributed to success of this case. Instead, the concluding takeaways were limited to learning what the standard protocol is.

There is undoubtedly much to learn from the study of Safety-I concepts. The less commonly focused on and emphasized Safety-II concepts, however, offers additional and different opportunities for learning and improvement, which remain disproportionately unexplored by the facilitators. Unfortunately, there are multiple examples of positive performance aspects in the videos that were mentioned by participants but then not discussed further. There were the expected “great job” and other reinforcing accolades of agreement that something “went well” but without subsequent exploration into why. For example, in one case, the facilitator commented that “communication was effective” and the team agrees but there are no follow-up comments of any specifics of what made it effective nor is there follow-up probing or comment by the facilitator. This example highlights the missed opportunity to explore and ask “what about it was effective?”; “what is effective communication?”; “was this communication similar to other cases you’ve been in and how so?”; “why was communication more effective in this case than other examples?”; or “what conditions set us up for effective communication and how can we replicate or promote them in the future?”. It is likely that members of that team agreed the communication was effective but that a team member or members may have been left wondering “why?”; “what about it was effective?”; or how to emulate it in the future (e.g., the debriefing missed an important opportunity to discuss communication best practices or communication tools). Furthermore, intentional discussion on positive communication and teamwork dynamics in the framework of a Safety-II lens requires facilitation and deeper questions and exploration of variability, flexibility, and specifics to ensure reproduction of success in future cases. It is also likely to help inform and promote productive, future discussions on improving communication in other cases when communication is not “effective.” It is crucial to discuss and learn from positive performance but Safety-II requires further intentional exploration, which may be guided by available Safety-II debriefing tools [22], questioning for example, “why did it go well?”; “what actions facilitated the good performance?”; “were there strategies used in this case to make work more efficient?” and ultimately “how can we learn from this success and reproduce or incorporate it into future performance?” Despite the numerous noted discussions of positive performance in the debriefing videos, there were relatively fewer explorations into why it occurred or how it might be reproduced in the future.

These examples highlight the broad opportunity in almost any debriefing to include debriefing focused on both Safety-I and Safety-II. As seen in this analysis, there is a common emphasis on exploration of negative performance and Safety-I. It is wonderful to acknowledge and point out examples of positive performance but we must do better to intentionally discuss these positive aspects, analyze why, and deepen learning from these aspects to ensure they are reproduced in the future. This is the gap that debriefing inclusive of Safety-II can bridge. And it is also important to note that the specific Safety-II concepts listed were assigned when the debriefings were analyzed for this study. None of the terms were explicitly named, taught about, or included in the discussion. Development of more common use of Safety-II language, frameworks, and tools to guide debriefings is another area of opportunity to support the inclusion of Safety-II exploration in future debriefings.

This study is limited by the small number of videos available for review, which may restrict the generalizability of the findings. These open-access healthcare debriefing videos were selected in efforts to assess Safety-I or Safety-II focus in these publicly available, instructional models of healthcare debriefing. Despite this constraint, however, the analysis serves as a valuable quantification of the disproportionate focus on negative performance exploration and Safety-I compared to Safety-II discussions in a small but specific subset of available material. This serves to highlight the need for further research with a broader dataset to enhance the depth of understanding and guide further tailored support for debriefing inclusive of Safety-II.

## Conclusion

Safety-I focuses heavily on errors, failures, and deviations from protocols in order to prevent future adverse outcomes; while Safety-II ensures that learning is not just about preventing failures but also focuses on adaptations, variability, and human capacities in order to reinforce resilience. Overall, patient safety science and many debriefing approaches tend to emphasize analysis of negative over positive performance and focus most heavily on Safety-I. Consequently, rich analysis and learning from Safety-II is underrepresented or excluded entirely. While there is always room for improvement and we must all strive to do the best we can, we are missing a major opportunity to build resilience if we don’t analyze the reasons why things go as positively as they do. Review of open-access video examples of healthcare debriefing demonstrates this phenomenon of disproportionate emphasis on both negative performance over positive performance and on exploration of Safety-I more than Safety-II. This highlights the opportunity for open-access examples of healthcare debriefing to include additional language and techniques that promote and role model inclusion of Safety-II discussion. Those designing such instructional videos should intentionally include debriefing of both Safety-I and Safety-II aspects of performance, as they are both important and complement one another. Future study on the impact of Safety-II debriefing should focus on context-specific promotion of quality and patient safety, as well as impact on participant wellbeing and overall debriefing culture.

## Data Availability

No datasets were generated or analysed during the current study.
